# The clinical efficacy and safety evaluation of ticagrelor for acute coronary syndrome in general ACS patients and diabetic patients: A systematic review and meta-analysis

**DOI:** 10.1371/journal.pone.0177872

**Published:** 2017-05-17

**Authors:** Qiutong Tan, Xin Jiang, Sichao Huang, Tiantian Zhang, Lin Chen, Siwen Xie, Enpan Mo, Jun Xu, Shaohui Cai

**Affiliations:** 1 College of Pharmacy, Jinan University, Guangzhou, P. R. China; 2 Medical Division, Renolit, Beijing, P.R. China; 3 Department of Pharmacy, Zhuhai People’s Hospital, Zhuhai, P. R. China; 4 Institution of Drug Clinical Trail, the First Affiliated Hospital of Jinan University, Guangzhou, P.R. China; University of Bologna, ITALY

## Abstract

**Objective:**

In this study, a systematic evaluation was conducted to estimate the efficacy and safety of ticagrelor for treating acute coronary syndrome (ACS) in general ACS patients and a diabetes mellitus (DM) group.

**Methods:**

A search of PubMed, Cochrane Central Register of Controlled Trials, Web of Science, CNKI databases was conducted to analyze relevant randomized controlled trails (RCTs) of ticagrelor treating ACS during 2007 to 2015. Article screening, quality accessing and data extracting was independently undertaken by two reviewers. A meta-analysis was performed to clarify the efficacy and safety of ticagrelor in general ACS patients, and a meta-regression analysis was taken to demonstrate the efficacy and safety of ticagrelor in DM patients compared with general ACS patients.

**Result:**

Twenty-two studies with 35004 participants were included. The meta-analysis result implicated that ticagrelor could: 1) reduce the incidence of the composite endpoint [OR = 0.83, 95%CI (0.77, 0.90), P<0.00001] and the incidence of myocardial infarction [OR = 0.81, 95%CI (0.74, 0.89), P = 0.0001]; 2) not statistically reduce the incidence of cardiovascular death, the incidence of stroke and the incidence of bleeding events; 3) increase the incidence of dyspnea [OR = 1.90, 95%CI (1.73, 2.08), P<0.00001] compared with clopidogrel. Meanwhile, compared with prasugrel, ticagrelor could 1) reduce the platelet reactivity of patients at maintenance dose [MD = -44.59, 95%CI (-59.16, -30.02), P<0.00001]; 2) not statistically reduce the incidence of cardiovascular death, the platelet reactivity of patients 6 hours or 8 hours after administration, or the incidence of bleeding events; 3) induce the incidence of dyspnea [OR = 13.99, 95%CI (2.58, 75.92), P = 0.002]. Furthermore, the result of meta-regression analysis implicated that there was a positive correlation between DM patients and the platelet reactivity of patients 6 hours and 8 hours after administration, but there was no obvious correlation between DM patients and general ACS patients in other endpoints.

**Conclusion:**

Ticagrelor could reduce the incidence of composite endpoint of cardiovascular death, myocardial infarction and stroke as well as platelet reactivity in DM patients with ACS, while not increasing the risk of bleeding. Because there are differences in platelet reactivity between DM patients and general ACS patients, we suggest that caution is needed when using ticagrelor in clinical applications.

## Introduction

Acute coronary syndrome (ACS) refers to a group of clinical conditions such as coronary atherosclerosis rupture, platelet aggregation and thrombosis. Platelet aggregation has a close relationship with the occurrence and development of ACS; thus, antiplatelet therapy is the most common treatment for ACS.

Second-generation thienopyridines (clopidogrel and prasugrel) are widely used in antiplatelet therapy. Clopidogrel is converted to its active metabolites in vivo by a 2-step process, and these active metabolites irreversibly inhibit the platelet P2Y12 adenosine diphosphate receptor [1, 2]. Therefore, clopidogrel is a prodrug, and its onset of action is relatively slow [3]. Moreover, 30% of patients show drug resistance to clopidogrel, which can induce a high risk of myocardial infarction recurrence and stent thrombosis [4]. Prasugrel is another antiplatelet drug with the same mechanism as clopidogrel. Its active metabolites are produced in a 1-step metabolic process; thus, its onset of action is shorter [5]. Furthermore, compared with clopidogrel, it has a series of advantages, such as greater efficacy and lower variability. However, it probably has an increased risk of bleeding, including fatal bleeding [6–8]. Given the limitations of these two widely used drugs, such as the delayed onset of action and variability of clopidogrel and prasugrel bleeding risk, additional studies were critical in developing efficient new P2Y12 receptor antagonists.

Ticagrelor (AZD6140) is the first reversibly binding oral P2Y12 receptor antagonist that blocks ADP-induced platelet aggregation. The discovery of ticagrelor began with adenosine triphosphate (ATP). The subsequent identification of a novel series of P2Y12 receptor antagonists and the exploitation of their SAR has been described. Modifications of the acidic side chain and purine core, in addition to experimentation with hydrophobic substituents, led to the development of a series of neutral molecules. Ultimately, the leading compound, AZD6140, was developed as a novel platelet aggregation inhibitor [9].

Unlike the thienopyridines, ticagrelor is not a prodrug and therefore does not require metabolic activation. It binds reversibly to the receptor and exhibits rapid onset and offset of action, which closely follows drug exposure levels [10]. The action mechanism of ticagrelor facilitates the rapid recovery of platelet function after drug withdrawal. Ticagrelor also has a stronger and more consistent effect than clopidogrel because its direct action does not require catabolite activation [11].

Several clinical studies have indicated that ticagrelor is superior to clopidogrel in reducing platelet reactivity, myocardial infarction, cardiovascular death, stroke and adverse events [12]. It may also reduce the incidence of clinical bleeding events compared with prasugrel [13, 14]. However, some authors have noted that the bleeding risk of the two treatments is not significantly different [15]. Therefore, further research is needed to estimate the safety of ticagrelor compared to the other two drugs.

During our analysis of clinical studies on ticagrelor, clopidogrel and prasugrel, we found that a significant proportion of patients with ACS have comorbid diabetes mellitus (DM). This result underscores the fact that DM is an important risk factor for ACS. DM enhances the risk of coronary and cerebrovascular diseases [16] and significantly increases the risk of major cardiovascular complications [17, 18]. Diabetic patients comprise a unique subpopulation within ACS, and the clinical effects of aspirin are different in diabetic patients than in other ACS patients. According to a meta-analysis by De Berardis G et al., the benefit of aspirin in DM patients is well below expectations, which may be explained by the rapid recovery of platelet reactivity in DM patients [19]. Although some clinical studies have started to focus on the clinical efficacy of ticagrelor in DM patients, a systematic review of the efficacy and safety of ticagrelor in DM patients with ACS has been lacking.

Therefore, in this article, we systematically evaluated the efficacy and safety of ticagrelor in DM patients with ACS compared with clopidogrel and prasugrel. The results may provide a guideline for more effective treatment of ACS in patients with DM.

## Materials and methods

### Search strategy and eligibility criteria for study selection

Randomized controlled trials comparing the clinical efficacy of ticagrelor and clopidogrel or prasugrel in treating ACS, published from 2007 to 2015, were screened for inclusion in this study. The PubMed, Cochrane Central Register of Controlled Trials, Web of Science, China National Knowledge Infrastructure (CNKI) databases were searched using the following terms: “ticagrelor or AZD6140 or Brilinta” and “clopidogrel or Plavix or prasugrel or CS-747 or LY 640315” and “acute coronary syndrome or stable coronary artery” and “randomized controlled trails”. The detailed search strategy was provided in S1 Fig.

Reviews and other relevant articles were also searched to identify all potential results. The citation lists of the retrieved articles were manually screened by the inclusion and exclusion criteria. The detail of inclusion criteria were as follows: 1) study designs were clearly described as clinical trials; 2) Ticagrelor was used as the experimental drug, and either clopidogrel or prasugrel was used as the positive control drug; 3) patients in the ticagrelor group were given a loading dose of 180 mg orally, followed by a maintenance dose of 90 mg twice a day; patients in the clopidogrel group were given a loading dose of 300 mg orally followed by a maintenance dose of 75mg; patients in the prasugrel group were given a loading dose of 60 mg orally, followed by a maintenance dose of 10 mg twice a day; and all patients were given aspirin 75–100mg per day unless they were intolerant; 4) the duration of treatment was less than 12 months; and 5) participants were suffering from ACS with unstable angina, non-ST segment elevation myocardial infarction or ST segment elevation myocardial infarction. In addition, exclusion criteria were as follows: 1) original studies were non-clinical trials; 2) the drug in the control group was neither clopidogrel nor prasugrel; 3) endpoints in the studies were not in accordance with the endpoints in this systematic review; 4) studies had insufficient data for analysis; and 5) the article is a review or letter.

### Endpoints of evaluation

The primary endpoint is a composite endpoint (containing the probability of any myocardial infarction, cardiovascular death or stroke). Secondary endpoints included the incidence of myocardial infarction (MI), cardiovascular death (CVD), stroke and the platelet reactivity. The endpoints in the safety evaluation included bleeding events and dyspnea.

### Data extraction

Two investigators (QT and SX) independently screened the articles and extracted the data from the included studies using standard data-abstraction forms. Disagreements were resolved by discussion with another reviewer (JX). Extracted data were transferred to Review Manager 5.2 for meta-analysis and Stata 12.0 for meta-regression analysis.

The following information was extracted from included studies: First author, year of publication, disease that participants suffered, intervention, efficacy outcomes and adverse events.

### Risk of bias analysis

Two reviewers (QT and SX) independently assessed the quality of the included studies according to the Cochrane risk of bias tool, which assesses the following six domains: selection bias, performance bias, detection bias, attrition bias, reporting bias and other bias [20].

### Statistical methods

The meta-analysis was prepared using Review Manager 5.2 software. The data extracted from the included studies were used to calculate odds ratios (OR), mean difference (MD) and 95% confidence intervals (CIs). A fixed-effects model was applied to the overall analysis and subgroup analysis if no heterogeneity was expected while a random-effects model was applied if heterogeneity was present. I^2^ (>50%) calculated by Review Manager 5.2 software were taken as the determinant of heterogeneity and P value (<0.05) was considered statistically significant. Publication bias was assessed with Harbord’s test (for dichotomous variables) or Egger’s test (for continuous variables), significant publication bias was indicated when P<0.05. These data were depicted in funnel plots. The meta-regression analysis, prepared using Stata 12.0 software, was used to evaluate the relationship between the DM patient proportion and the efficacy and safety endpoints.

## Result

### Description of the studies

**Fig 1:**
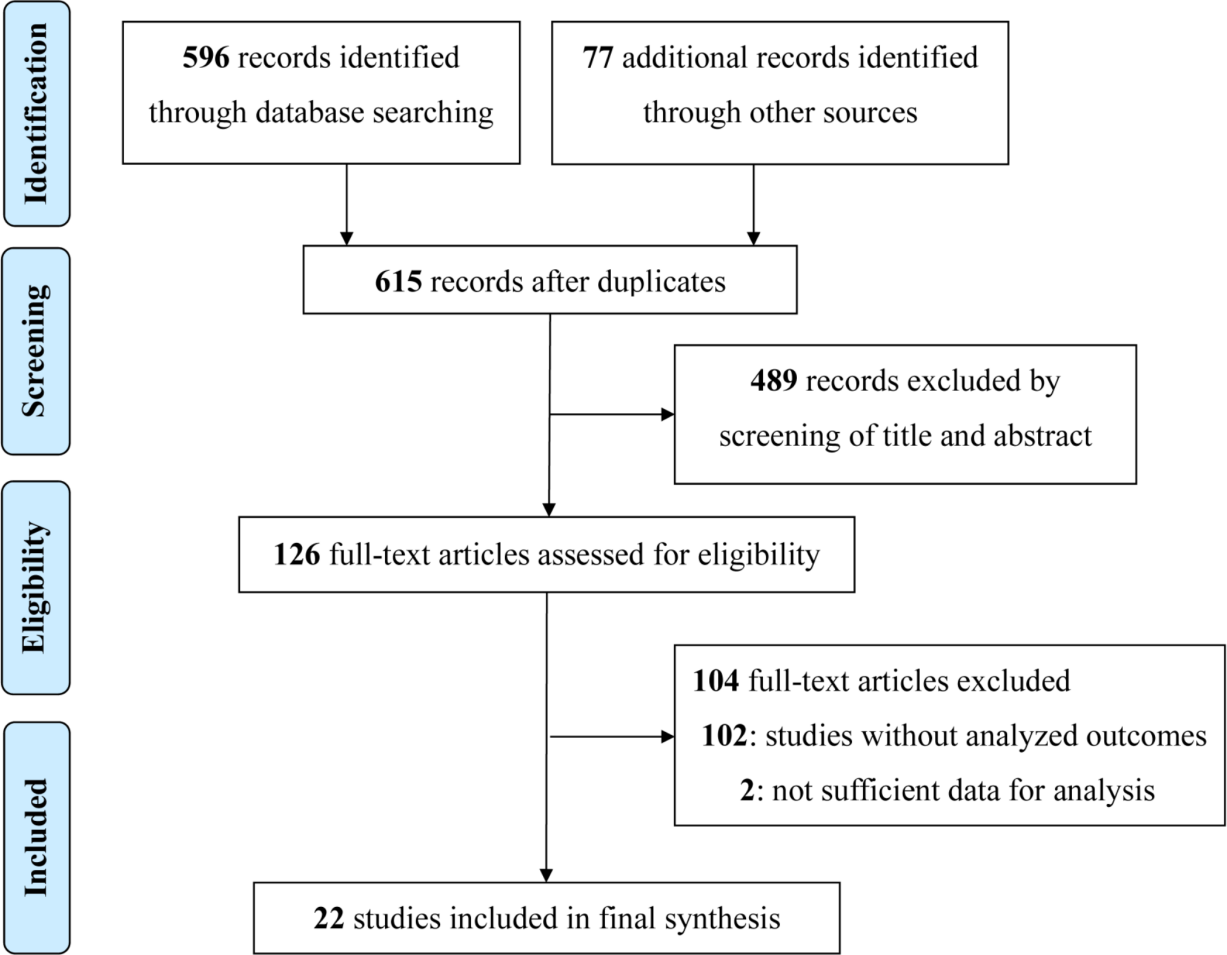
Process and results of study selection.

A detailed description of study screening is illustrated in Fig 1. A total of 596 studies were identified through database searches; 77 additional records were identified through other sources. After removing any duplicates, 615 studies remained. Among them, 489 studies were excluded by screening of title and abstract, 102 studies were excluded for lack of analyzed outcomes, and 2 studies were excluded for insufficient data for analysis. Twenty-two studies [1, 21–41] covering a total of 35,004 participants were finally included in this systematic review.

The basic characteristics of the included studies are listed in Table 1. The analysis included patients with acute coronary syndrome, such as coronary artery disease (CAD), non-ST segment elevation myocardial infarction (NSTEMI), ST segment elevation myocardial infarction (STEMI) and unstable angina. Among the 22 included studies, 14 studies compared the clinical efficacy of ticagrelor and clopidogrel, while 8 studies compared ticagrelor to prasugrel. The outcomes evaluated between ticagrelor groups and clopidogrel or prasugrel groups varied across the included studies. Pooled outcomes contained incidence of the composite endpoint, platelet reactivity, the incidence of bleeding events, the incidence of myocardial infarction, the incidence of cardiovascular death, the incidence of stroke and the incidence of dyspnea.

**Table 1:** Characteristics of included studies.

No.	Year	Patients (DM Patients)	Disease	Intervention	Outcomes ^a^
1 [21]	2010	13408 (3109)	ACS	Ticagrelor vs Clopidogrel	1, 3, 4, 5, 6
2 [22]	2013	100 (100)	ACS	Ticagrelor vs Prasugrel	2, 3, 5
3 [23]	2014	405 (108)	ACS with CABG	Ticagrelor vs Clopidogrel	1, 3
4 [1]	2013	30 (30)	ACS with DM	T-P vs P-T	2, 3, 4, 7
5 [24]	2014	58 (32)	CAD with DM	Ticagrelor vs Clopidogrel	2
6 [25]	2014	60 (23)	APC in ACS	Ticagrelor vs Clopidogrel	2, 3, 7
7 [26]	2013	159 (62)	ACS	Ticagrelor vs Clopidogrel	2
8 [27]	2007	984 (241)	NSTE-ACS	Ticagrelor vs Clopidogrel	1, 3, 4, 5, 6, 7
9 [28]	2009	18624 (4662)	ACS	Ticagrelor vs Clopidogrel	1, 3, 4, 5, 6, 7
10 [29]	2014	160 (32)	ACS	Ticagrelor vs Clopidogrel	1, 3, 4, 5, 6, 7
11 [30]	2014	63 (19)	ACS	Ticagrelor vs Clopidogrel	3, 4
12 [31]	2015	114 (36)	ACS	Ticagrelor vs Prasugrel	3
13 [32]	2013	50 (9)	STEMI	Ticagrelor vs Prasugrel	2, 4, 5, 6, 7
14 [33]	2014	98 (31)	Stable CAD	Ticagrelor vs Prasugrel	2
15 [34]	2015	55 (5)	STEMI	Ticagrelor vs Prasugrel	2, 3, 5
16 [35]	2009	101 (22)	Stable CAD	Ticagrelor vs Clopidogrel	2, 3, 7
17 [36]	2014	20 (6)	ACS	Ticagrelor vs Prasugrel	2
18 [37]	2012	44 (10)	ACS & HTPR	T-P vs P-T	2, 3, 7
19 [38]	2015	40 (21)	Stable CAD	T-C vs C-T	2, 7
20 [39]	2015	157 (157)	STEMI with DM	Ticagrelor vs Clopidogrel	3, 4, 5, 7
21 [40]	2015	120 (80)	ACS	Ticagrelor vs Clopidogrel	7
22 [41]	2015	154 (154)	STEMI with DM	Ticagrelor vs Clopidogrel	3, 5, 7

Characteristics of included studies

^a^ Outcomes: 1 incidence of composite endpoint; 2 platelet reactivity; 3 incidence of bleeding events; 4 incidence of myocardial infarction; 5 incidence of cardiovascular death; 6 incidence of stroke; 7 incidence of dyspnea

**Fig 2:**
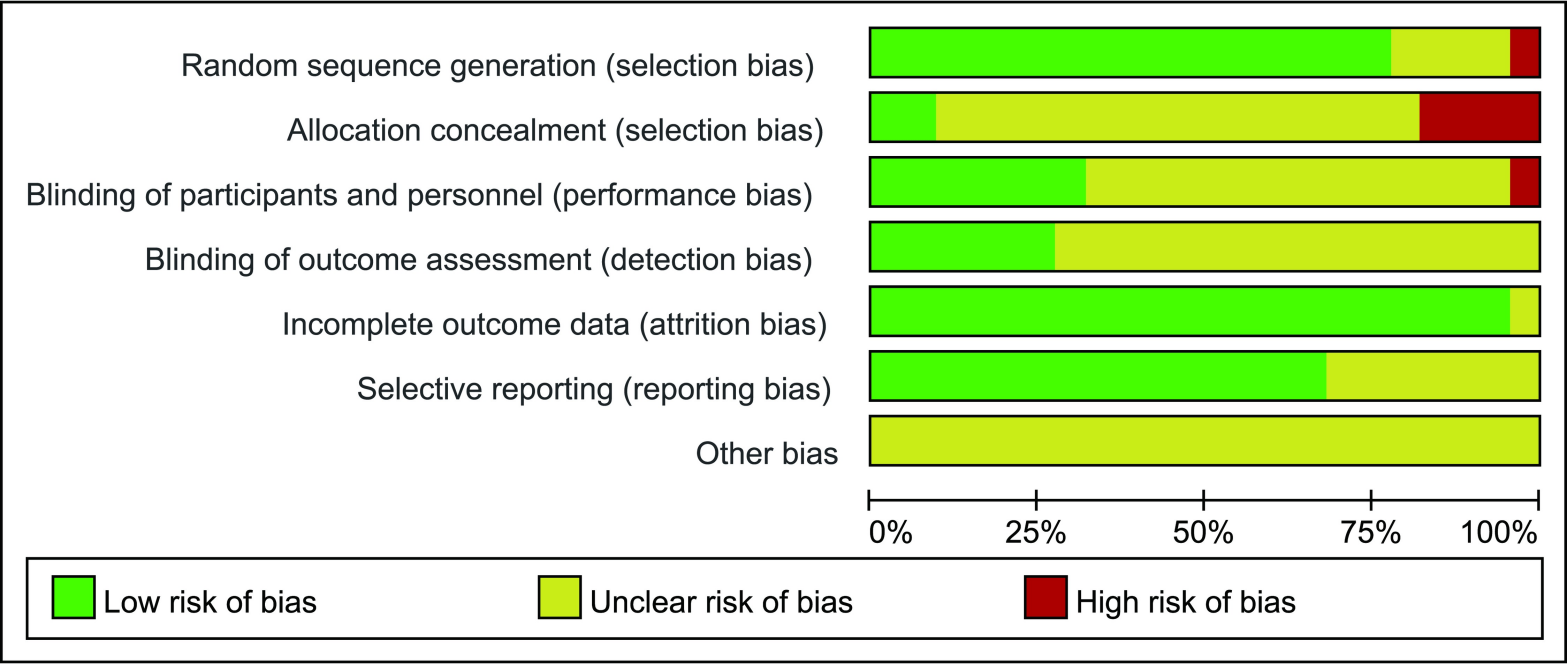
Risk of bias graph.

**Fig 3:**
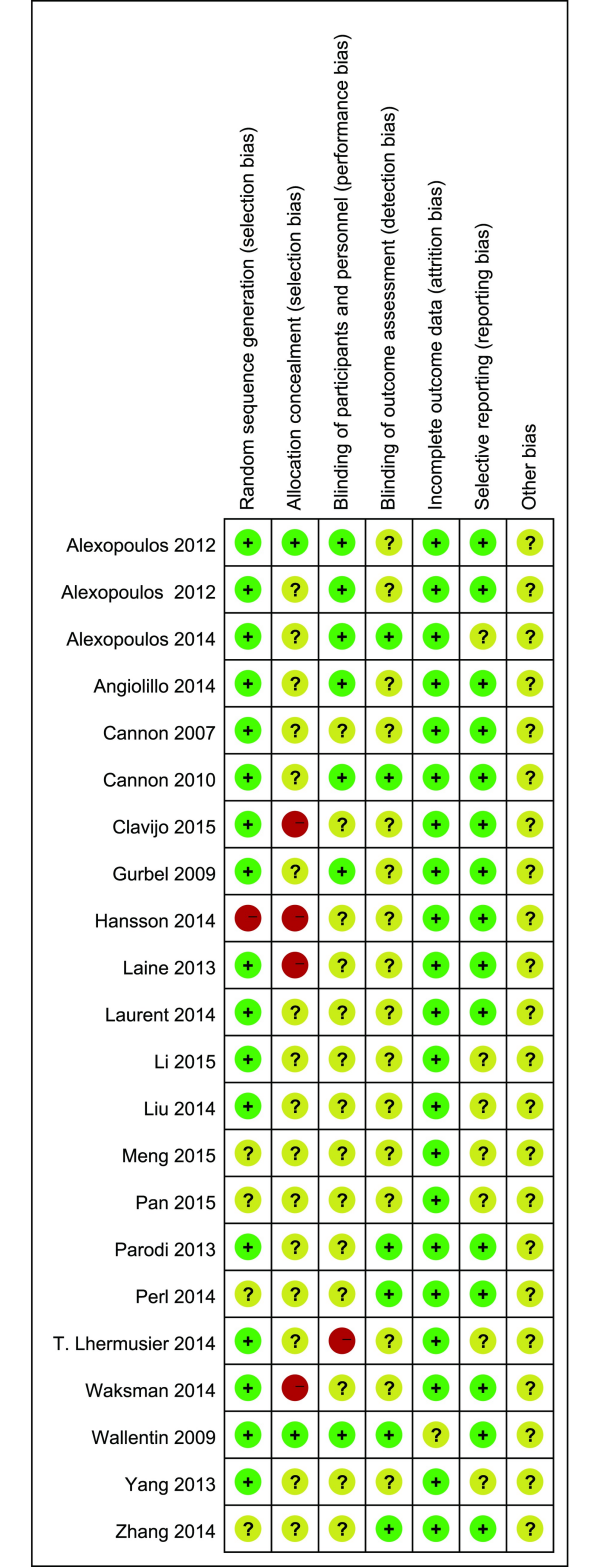
Risk of bias summary.

The Cochrane risk of bias tool was used to measure the quality of the included studies, and the results are shown in Figs 2 and 3. Most of the included studies describe the detail of random sequence generation, incomplete outcome data and selective reporting. Some studies did not mention allocation concealment, blinding of participants and personnel or random sequence generation. The other indexes of bias usually lacked specific description in the included clinical studies.

#### Incidence of composite endpoint

**Fig 4:**
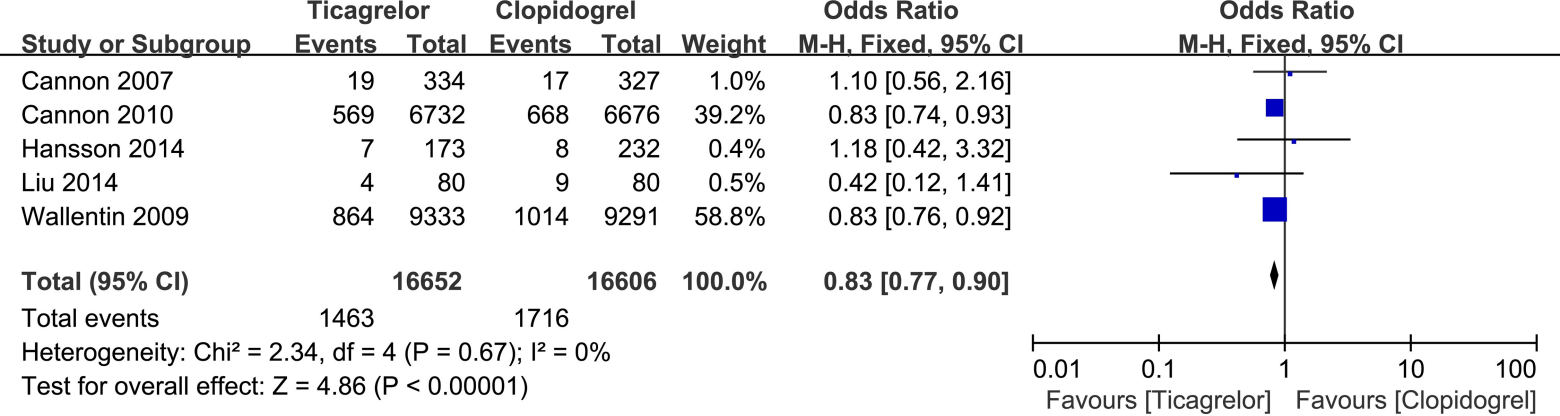
Forest plot of incidence of composite endpoint in ticagrelor and clopidogrel group.

**Fig 5:**
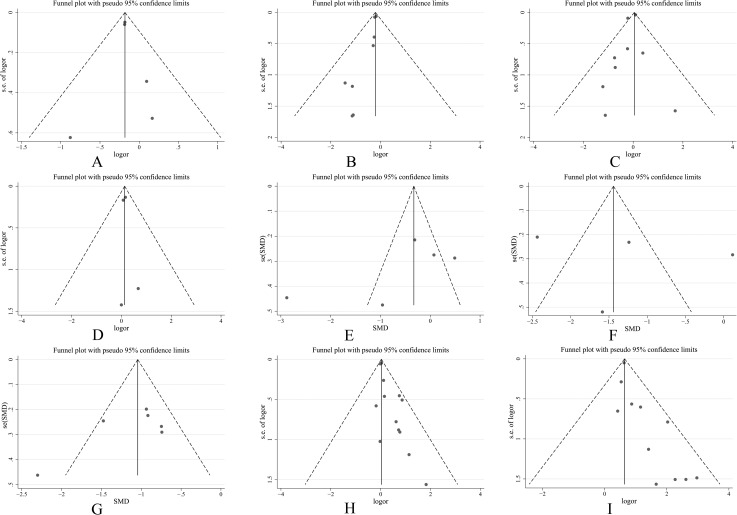
Funnel plots for the assessment of publication bias. (A) Incidence of composite endpoint; (B) Incidence of myocardial infarction; (C) Incidence of cardiovascular death; (D) Incidence of stroke; (E) Platelet reactivity 6 hours after administration; (F) Platelet reactivity 8 hours after administration; (G) Platelet reactivity at maintenance dose; (H) Incidence of bleeding events; (I) Incidence of dyspnea.

**Fig 6:**
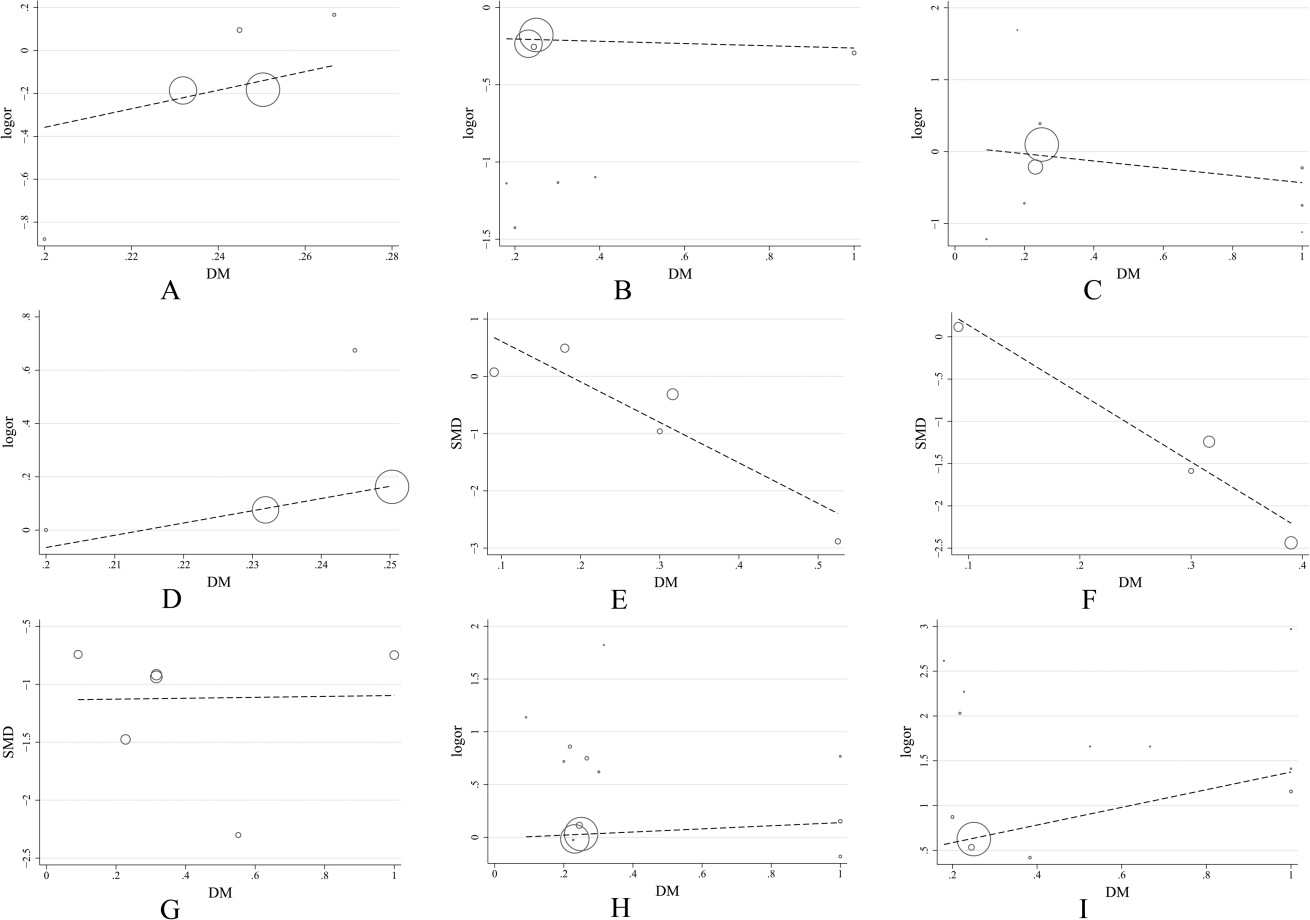
Scatter plots of meta-regression analysis. (A) Incidence of composite endpoint; (B) Incidence of myocardial infarction; (C) Incidence of cardiovascular death; (D) Incidence of stroke; (E) Platelet reactivity 6 hours after administration; (F) Platelet reactivity 8 hours after administration; (G) Platelet reactivity at maintenance dose; (H) Incidence of bleeding events; (I) Incidence of dyspnea.

As far as general ACS patients are concerned, 5 studies, including a total of 33,258 patients, assessed the incidence of the composite endpoint. Low heterogeneity was shown among the studies [P = 0.67, I2 = 0%], and according to the fixed-effects model, the incidence of the composite endpoint in the ticagrelor group was significantly lower than the incidence in the clopidogrel group [OR = 0.83, 95%CI (0.77, 0.90), P<0.00001] (Fig 4). The funnel plot did not demonstrate publication bias (Pharbord = 0.868) (Fig 5A). A meta-regression analysis was conducted to evaluate the relationship between the DM patient proportion and the incidence of the composite endpoint. The result is shown in Fig 6A, which indicates that the incidence of the composite endpoint demonstrated no significant relationship with DM patient proportion (P = 0.532).

#### Incidence of myocardial infarction

**Fig 7:**
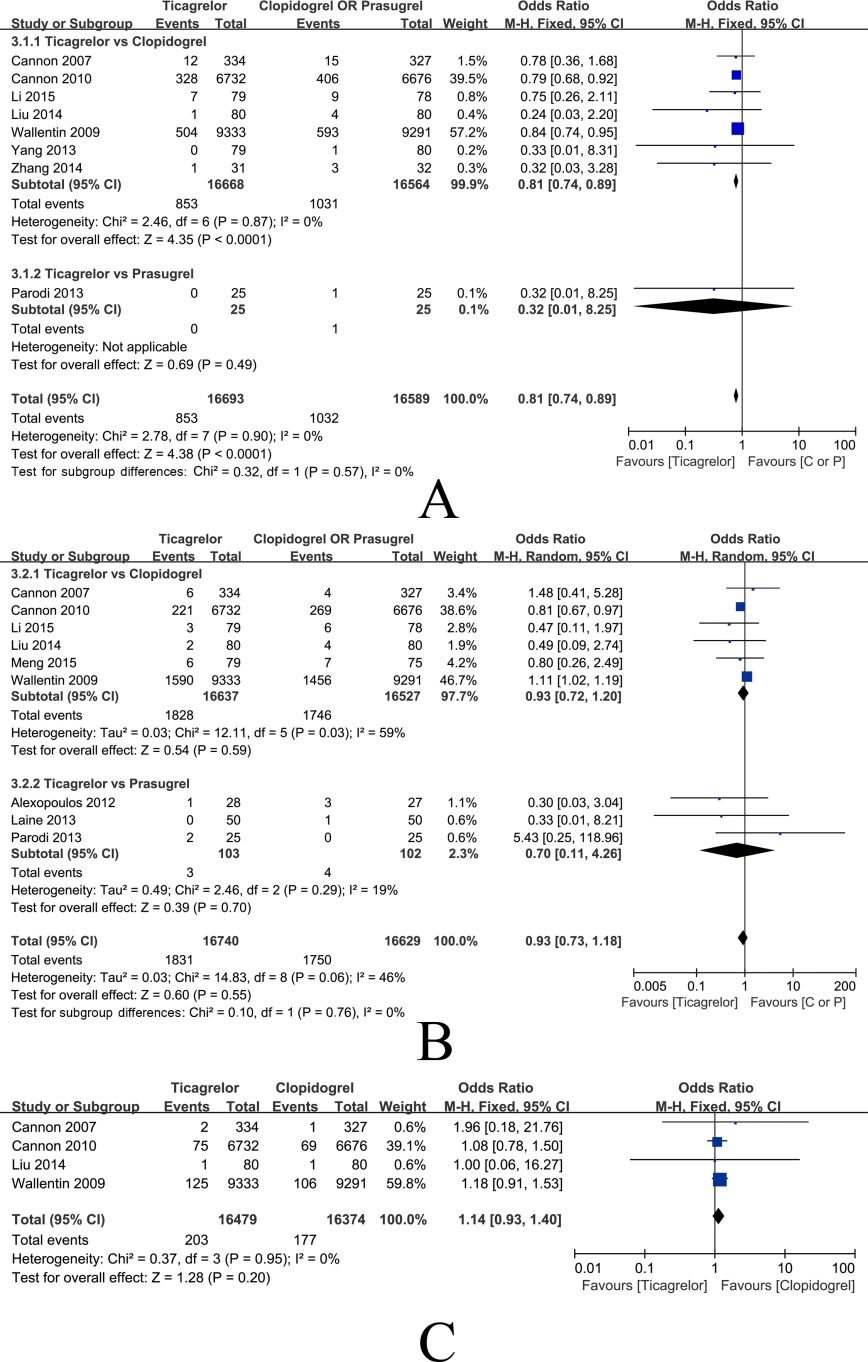
Forest plots of MI, CVD and stoke. (A) Incidence of myocardial infarction; (B) Incidence of cardiovascular death; (C) Incidence of stroke.

8 studies in general ACS patients comprising 33,282 patients were eligible for the final analysis. Low heterogeneity was shown among the studies [P = 0.90, I2 = 0%] and meta-analysis by fixed-effects model indicates that the incidence of myocardial infarction in the ticagrelor group is significantly lower than the incidence in the clopidogrel and prasugrel groups [OR = 0.81, 95%CI (0.74, 0.89), P<0.0001]. Subgroup analysis showed that the incidence of myocardial infarction in the ticagrelor group was significantly lower than the incidence in the clopidogrel group [OR = 0.81, 95%CI (0.74, 0.89), P = 0.0001]. One study on prasugrel showed that the incidences of myocardial infarction were 0.0% and 3.9% in the ticagrelor and prasugrel groups, respectively (Fig 7A). The results showed signs of publication bias, as determined by the funnel plot in Fig 5B (Pharbord = 0.005). Meta-regression revealed that the results demonstrate no significant relationship between DM patient proportion and the occurrence of myocardial infarction (P = 0.920) (Fig 6B).

#### Incidence of cardiovascular death

Nine studies in general ACS patients comprising 33,369 patients were eligible for the final analysis. Heterogeneity was shown among the studies [P = 0.06, I2 = 46%], and the meta-analysis by random-effects model indicated that the incidence of cardiovascular death in the ticagrelor group was not significantly different from the incidence in the clopidogrel and prasugrel groups [OR = 0.93, 95%CI (0.73, 1.18), P = 0.55]. Subgroup analysis showed that the ticagrelor group had no significant difference form the clopidogrel [OR = 0.93, 95%CI (0.72, 1.20), P = 0.59] and prasugrel groups [OR = 0.70, 95%CI (0.11, 4.26), P = 0.70] (Fig 7B).The funnel plot in Fig 5C reveals that the results did not demonstrate publication bias (Pharbord = 0.282) and no significant relationship was found between DM patient proportion and the incidence of cardiovascular death by meta-regression (P = 0.446), as shown in Fig 6C.

#### Incidence of stroke

Four studies in general ACS patients comprising 32,853 patients were eligible for the final analysis. Low heterogeneity was shown among the studies [P = 0.95, I2 = 0%] and meta-analysis by fixed-effects model indicates that the incidence of stroke in the ticagrelor group was not significantly different from the incidence in the clopidogrel group [OR = 1.14, 95%CI (0.93, 1.40), P = 0.20] (Fig 7C). The funnel plot in Fig 5D indicates no publication bias (Pharbord = 0.687) and no significant relationship was found between DM patient proportion and the occurrence of stroke by meta-regression (P = 0.716), as shown in Fig 6D.

#### Platelet reactivity after 6 hours

**Fig 8:**
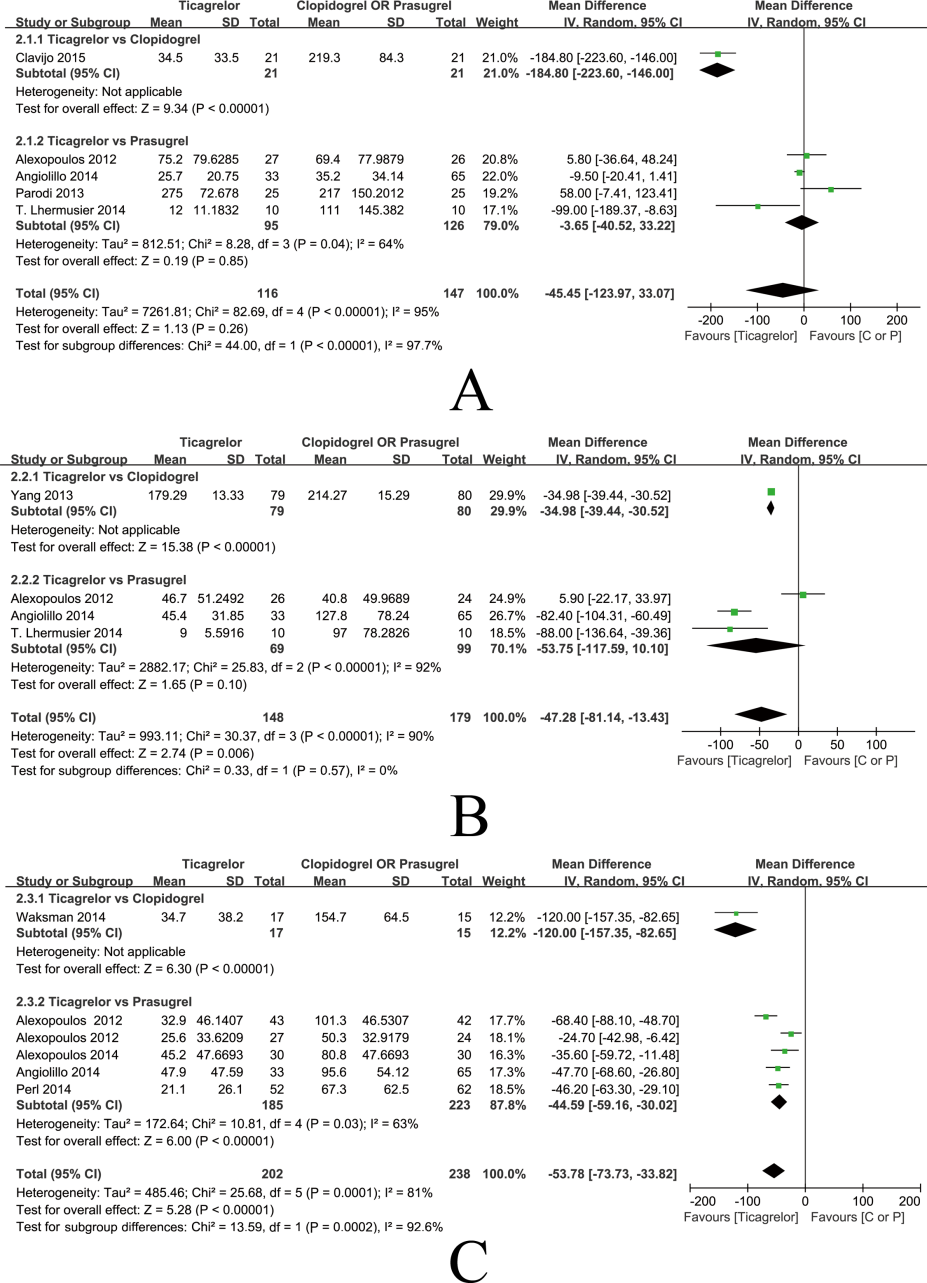
Forest plots of platelet reactivity. (A) Platelet reactivity 6 hours after administration; (B) Platelet reactivity 8 hours after administration; (C) Platelet reactivity at maintenance dose.

Five studies comprising 263 patients assessed platelet reactivity 6 hours after the administration of therapy in general ACS patients. High heterogeneity was shown among the studies [P<0.00001, I2 = 95%]. Thus, the random-effects model was used to evaluate the data, which showed that platelet reactivity 6 hours after the administration of therapy was not significantly different in the ticagrelor and control groups [MD = -45.45, 95%CI (-123.97, 33.07), P = 0.26] (Fig 8A). Further subgroup analysis showed no statistically significant difference between the ticagrelor group and the prasugrel group [MD = -3.65, 95%CI (-40.52, 33.22), P = 0.85]. One study on clopidogrel showed that both ticagrelor and clopidogrel inhibit platelet reactivity 6 hours after administration; the PRU values were 34.5 and 219.3, respectively (Fig 8A). No publication bias was observed, as determined by the funnel plot shown in Fig 5E (Pegger = 0.256). Further meta-regression analysis was conducted to evaluate the correlation between DM patient proportion and platelet reactivity 6 hours after administration of therapy. The results are shown in Fig 6E, which indicates that platelet reactivity 6 hours after administration demonstrates a significant relationship with DM patient proportion (P = 0.044).

#### Platelet reactivity after 8 hours

Four studies comprising 327 patients assessed platelet reactivity 8 hours after the administration of therapy in general ACS patients. Heterogeneity was shown among the studies [P<0.00001, I2 = 90%] and the random-effects model indicated that platelet reactivity 8 hours after the administration ticagrelor was significantly lower than clopidogrel and prasugrel [MD = -47.28, 95%CI (-81.14, -13.43), P = 0.006]. Subgroup analysis showed that there was no significant difference in the inhibition of platelet reactivity in the ticagrelor group 8 hours after administration of therapy compared to the prasugrel group [MD = -53.57, 95%CI (-117.59, 10.10), P = 0.10]. One study on clopidogrel showed that both ticagrelor and clopidogrel inhibited platelet reactivity 8 hours after administration; the PRU were 179.29 and 214.27, respectively (Fig 8B). The funnel plot in Fig 5F indicates no publication bias (Pegger = 0.679). Meta-regression analysis demonstrated that platelet reactivity 8 hours after the administration of therapy shows a significant relationship with DM patient proportion (P = 0.045) (Fig 6F).

#### Platelet reactivity at maintenance dose

Six studies comprising 440 patients assessed platelet reactivity at maintenance doses in general ACS patients. Heterogeneity was shown among the studies [P = 0.0001, I2 = 81%], and the random-effects model indicated that platelet reactivity in the ticagrelor group was significantly lower than platelet reactivity in the clopidogrel and prasugrel groups [MD = -53.78, 95%CI (-73.73, -33.82), P<0.00001]. Subgroup analysis showed that the ticagrelor group demonstrated less platelet reactivity than the prasugrel group [MD = -44.59, 95%CI (-59.16, -30.02), P<0.00001]. One study on clopidogrel showed that both ticagrelor and clopidogrel inhibit platelet reactivity at maintenance doses; the PRU were 34.7 and 154.7, respectively (Fig 8C). The funnel plot in Fig 5G indicates no publication bias (Pegger = 0.222). Furthermore, meta-regression analysis shows no significant relationship between DM patient proportion and platelet reactivity at maintenance doses (P = 0.965) (Fig 6G).

#### Incidence of bleeding events

**Fig 9:**
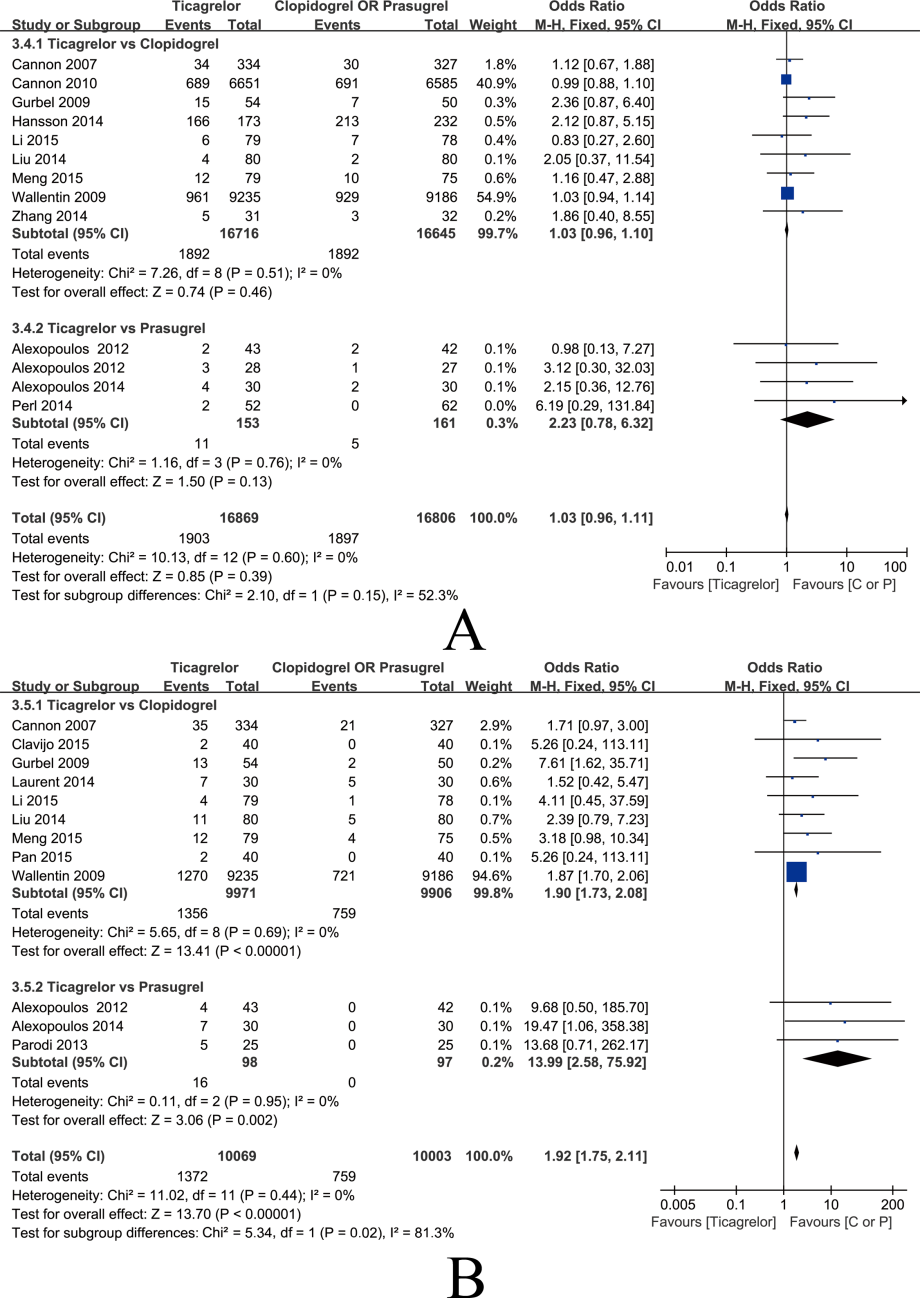
Forest plots of safety endpoint. (A) Incidence of bleeding events; (B) Incidence of dyspnea.

The data from 13 studies comprising 33,675 general ACS patients were eligible for incidence of bleeding events analysis and subgroup analysis. As shown in Fig 9A, low heterogeneity was shown among the studies [P = 0.60, I2 = 0%] and the pooled outcome of the fixed-effects model indicated that the incidence of bleeding events in the ticagrelor group was not significantly different from the incidence in the clopidogrel and prasugrel groups [OR = 1.03, 95%CI (0.96, 1.11), P = 0.39]. Subgroup analysis showed that the ticagrelor group had no significant difference from the clopidogrel [OR = 1.03, 95%CI (0.96, 1.10), P = 0.46] and prasugrel groups [OR = 2.23, 95%CI (0.78, 6.32), P = 0.13]. Signs of publication bias were observed, as determined by the funnel plot shown in Fig 5H (Pharbord = 0.003). Furthermore, no significant relationship was found between DM patient proportion and the incidence of bleeding by meta-regression (P = 0.744) (Fig 6H).

#### Incidence of dyspnea

Twelve studies comprising 20,072 general ACS patients were applicable for incidence of dyspnea analysis and subgroup analysis. As indicated in Fig 9B, low heterogeneity was shown among the studies [P = 0.44, I2 = 0%], and the meta-analysis by fixed-effects model indicated that the incidence of dyspnea in the ticagrelor group was significantly higher than the incidence in the clopidogrel and prasugrel groups [OR = 1.92, 95%CI (1.75, 2.11), P<0.00001]. Subgroup analysis showed that the incidence of dyspnea in the ticagrelor group was significantly higher than the incidence in the clopidogrel [OR = 1.90, 95%CI (1.73, 2.08), P<0.00001] and prasugrel groups [OR = 13.99, 95%CI (2.58, 75.92), P = 0.002]. The funnel plot in Fig 5I shows that the results demonstrated publication bias (Pharbord = 0.02); meta-regression revealed that the results did not demonstrate a significant relationship between DM patient proportion and the incidence of dyspnea (P = 0.160) (Fig 6I).

## Discussion

Ticagrelor is a novel reversible platelet inhibitor that is notable for its superior clinical efficacy and safety [42]. The results of our preliminary research show that DM patients may represent a high-risk population for angiocardiopathy and the clinical efficacy of ticagrelor in this group differs from the overall cohort. Thus, it was important to evaluate the efficacy and safety of ticagrelor in DM patients. In this study, we conducted a systematic evaluation to assess the efficacy and safety of ticagrelor in general ACS patients and DM patients who were suffering from ACS.

Although the 22 included RCTs comprise 35,004 participants, the various studies focused on different clinical indexes. Therefore, the primary endpoint was the incidence of the composite endpoint (5 studies, 33,258 cases, containing the probability of any myocardial infarction, cardiovascular death or stroke). The secondary endpoints were the incidence of myocardial infarction (8 studies, 33282 cases), the incidence of cardiovascular death (9 studies, 33369 cases), the incidence of stroke (4 studies, 32853 cases) and platelet reactivity (11 studies, 916 cases). The incidence of bleeding events (13 studies, 33675 cases) and the incidence of dyspnea (12 studies, 20072 cases) were used as safety endpoints.

Dosing is a crucial tissue in our research, and the conventional dosage of ticagrelor (90 mg bid) used in most of the clinical trials is based on the PLATO study (a phase III clinical study). Dosing in the PLATO study was based on the pharmacodynamics, efficacy and safety data demonstrated in the phase II study, these results indicated that a dose of 90 mg is well tolerated, acceptably safe and maximizes the inhibition of platelet aggregation [15]. Furthermore, higher doses of ticagrelor were not recommended, because the greatest suppression of platelet aggregation was seen at a dose of 90 mg. A lower dose of ticagrelor was not recommended because at lower doses the variability of platelet aggregation inhibition is more pronounced. In light of these two findings, we selected a dosage of 90 mg bid ticagrelor as an inclusion criterion for our study.

The duration of dual antiplatelet therapy also has an impact on efficacy and safety. In our study, the duration of antiplatelet therapy in the included studies was less than 12 month. However, the research of Fabrizio D’Ascenzo [43] noted that long-term usage (12 months or more) of dual antiplatelet therapy may increase the risk of bleeding events. Therefore, in this meta-analysis, the incidence of bleeding events was considered an important endpoint for the safety investigation of ticagrelor in general ACS and DM patients.

In this research, ticagrelor reduced the incidence of the composite endpoint in general ACS patients compared to clopidogrel, and these results were in accordance with the conclusions drawn in both the PLATO study [21] [9.0% vs 10.7%, 95%CI (0.75, 0.94), P = 0.0025] and in Wallentin’s research [28] [9.8% vs 11.7%, 95%CI (0.77, 0.92), P<0.001]. Both demonstrated that ticagrelor may significantly reduce the rate of myocardial infarction, death from vascular causes and the incidence of stroke.

As for secondary endpoints, ticagrelor may reduce the incidence of myocardial infarction compared to clopidogrel and prasugrel [OR = 0.81, 95%CI (0.74, 0.89), P<0.0001]. Ticagrelor did not statistically reduce the incidence of cardiovascular death compared to either clopidogrel or prasugrel [OR = 0.93, 95%CI (0.73, 1.18), P = 0.55]. Furthermore, ticagrelor did not significantly reduce the incidence of stroke compared to clopidogrel [OR = 1.14, 95%CI (0.93, 1.40), P = 0.20]. According to the research of Thibault Lhermusier, ticagrelor showed stronger inhibition of platelet reactivity in ACS patients compared to prasugrel [MD: -42.5, 95% CI: -62.9, -21.9] and was more effective than regular doses [MD: -159.7, 95% CI: -182.6, -136.6] or high doses [MD: -125.5, 95% CI: -154.9, -96.4] of clopidogrel [12]. The results of this study further supported the evidence presented above. Compared with clopidogrel and prasugrel, ticagrelor did not significantly reduce platelet reactivity after 6 hours [MD = -45.45, 95%CI (-123.97, 33.07), P = 0.26], but a significant reduction of platelet reactivity was found in the ticagrelor group after 8 hours [MD = -47.28, 95%CI (-81.14, -13.43), P = 0.006] and during the period of maintenance dosing [MD = -53.78, 95%CI (-73.73, -33.82), P<0.00001]. The results above demonstrate that the efficacy of ticagrelor is superior to the efficacy of clopidogrel or prasugrel in long-term treatment.

Ticagrelor did not statistically reduce the incidence of bleeding events compared with clopidogrel and prasugrel [OR = 1.03, 95%CI (0.96, 1.11), P = 0.39]. These results were also supported by the PRAGUE-18 Study (Zuzana Motovska, 2016) [44], which noted the head-to-head comparison of prasugrel and ticagrelor failed to show that one was more effective than the other in preventing bleeding events, including TIMI and BARC bleeding events. Furthermore, in Chirag’s research [45], ticagrelor showed a non-significant increase in TIMI major bleeding [OR = 1.14, 95%CI (0.74, 1.75), P = 0.10] and TIMI major/minor bleeding [OR = 1.07, 95%CI (0.97, 1.18), P = 0.89] compared with clopidogrel. These results were in accordance with our research.

Moreover, it is also worth noting that ticagrelor can significantly increase the incidence of dyspnea compared with clopidogrel and prasugrel. It may be that ticagrelor causes an increase in the endogenous adenosine concentration [46, 47] and the inhibition of P2Y_12_ on sensory neurons [48].

Based on the above results, meta-regression analysis was used to describe the difference in efficacy and safety of ticagrelor between diabetes mellitus patients and general ACS patients. There was no difference between general ACS patients and DM patients in reducing the incidence of the composite endpoint in the ticagrelor group. Meanwhile, the incidences of myocardial infarction, cardiovascular death and stroke showed no significant differences between general ACS patients and DM patients. However, the period of platelet reactivity recovery in DM patients was shorter than that of general ACS patients, which probably contributed to the higher platelet reactivity of DM patients with ACS. Nevertheless, in long-term usage, a non-significant difference in platelet reactivity was found between DM patients and general ACS patients. Furthermore, a non-significant difference was found between DM patients and general ACS patients in the incidence of bleeding events and dyspnea.

Our meta-regression analysis was also supported by the subgroup analysis of the PLATO study (4,662 patients with pre-existing DM and 13,951 patients without DM), which showed that the benefits and risks of ticagrelor in DM patients coincided with the outcomes of the cohort. Moreover, ticagrelor reduced the composite endpoints of cardiovascular death, myocardial infarction or stroke, and all-cause death in DM patients, without increasing the risk of bleeding. Low heterogeneity was present in general ACS and DM patients [2].

Nevertheless, it was noted that potential publication bias might exist in our study because it was a literature-based analysis, and a large proportion of the included publications showed positive results.

In conclusion, according to the existing research, ticagrelor exhibits superior clinical efficacy and safety than either clopidogrel or prasugrel in treating ACS. In the subgroup of DM patients, the clinical efficacy and safety of ticagrelor showed no obvious difference. However, the platelet reactivity of DM patients should be monitored in the treatment of ACS. Furthermore, more multi-center RCTs are required to ensure the reliability of these data and guide clinical practice.

## Supporting information

S1 File PRISMA 2009 checklist. (PDF)

S1 Fig Search strategy of this study. (TIF)

## Abbreviations

ACS: acute coronary syndrome
DM: diabetes mellitus
RCTs: randomized controlled trails
OR: odds ratios
MD: mean difference
CIs: 95% confidence intervals
CAD: coronary artery disease
NSTEMI: non-ST segment elevation myocardial infarction
STEMI: ST segment elevation myocardial infarction
