# Supplementary figures and images for: The clinical efficacy and safety evaluation of ticagrelor for acute coronary syndrome in general ACS patients and diabetic patients: A systematic review and meta-analysis
Source: PLoS One. 2017 May 17;12(5):e0177872. doi: 10.1371/journal.pone.0177872 (PMC5435320; doi:10.1371/journal.pone.0177872)

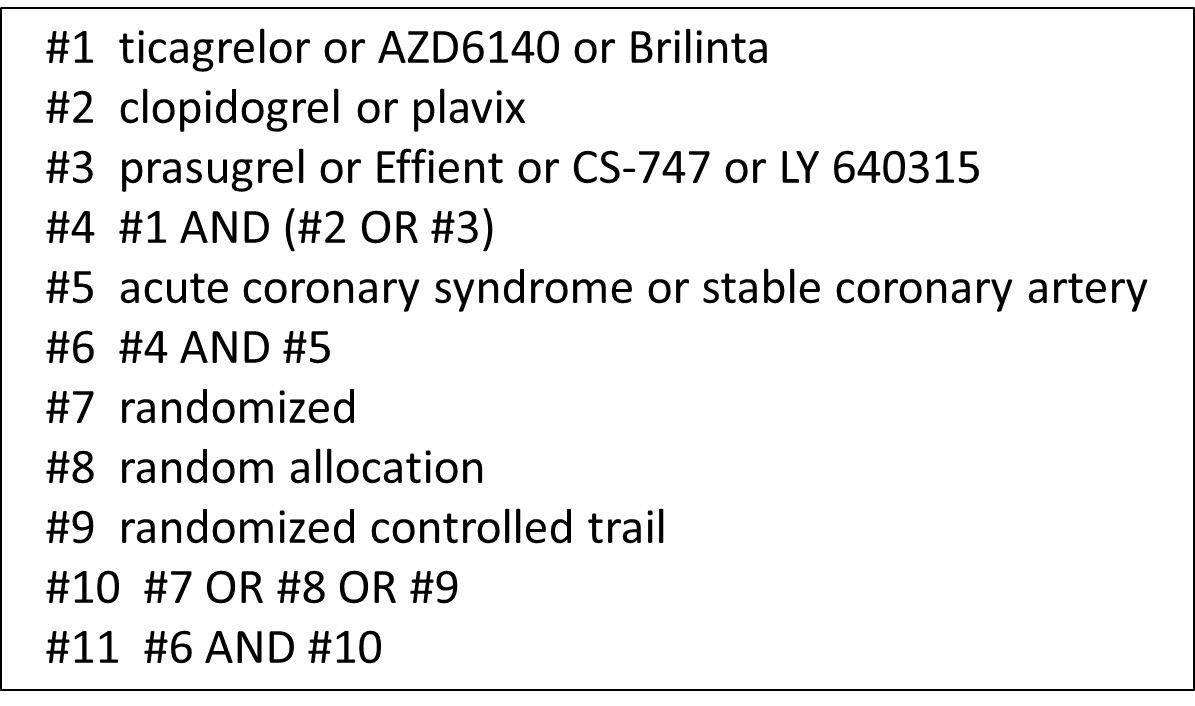

Supplement: S1 Fig — (TIF) [file pone.0177872.s002.tif]
